# Microbial Community Composition in Explanted Cystic Fibrosis and Control Donor Lungs

**DOI:** 10.3389/fcimb.2021.764585

**Published:** 2022-03-16

**Authors:** Gisli G. Einarsson, Bart M. Vanaudenaerde, Christopher D. Spence, Andrew J. Lee, Mieke Boon, Geert M. Verleden, J. Stuart Elborn, Lieven J. Dupont, Dirk Van Raemdonck, Deirdre F. Gilpin, Robin Vos, Stijn E. Verleden, Michael M. Tunney

**Affiliations:** ^1^ Halo Research Group, School of Medicine, Dentistry and Biomedical Sciences, Queen’s University Belfast, Belfast, United Kingdom; ^2^ Leuven Lung Transplant Unit, Department of Chronic Diseases, Metabolism and Ageing, KU Leuven, Leuven, Belgium; ^3^ Halo Research Group, School of Pharmacy, Queen’s University Belfast, Belfast, United Kingdom; ^4^ Department of Pediatics, Cystic Fibrosis Center, UZ Leuven, Leuven, Belgium; ^5^ Antwerp Surgical Training, Anatomy and Research Centre (ASTARC), University of Antwerp (UA), Wilrijk, Belgium; ^6^ Department of Thoracic & Vascular Surgery, University Hospital Antwerp (UZA), Edegem, Belgium; ^7^ Department of Pneumology, University Hospital Antwerp (UZA), Edegem, Belgium

**Keywords:** microbiota (16S rRNA), cystic fibrosis, lung explant, disease, health

## Abstract

To date, investigations of the microbiota in the lungs of people with Cystic Fibrosis (PWCF) have primarily focused on microbial community composition in luminal mucus, with fewer studies observing the microbiota in tissue samples from explanted lung tissue. Here, we analysed both tissue and airway luminal mucus samples extracted from whole explanted lungs of PWCF and unused donor lungs. We determined if the lung microbiota in end-stage CF varied within and between patients, was spatially heterogeneous and related to localized structural damage. Microbial community composition was determined by Illumina MiSeq sequencing and related to the CF-Computed Tomography (CT) score and features of end-stage lung disease on micro-CT. Ninety-eight CF tissue (n=11 patients), 20 CF luminal mucus (n=8 patients) and 33 donor tissue (n=4 patients) samples were analysed. Additionally, we compared 20 paired CF tissue and luminal mucus samples that enabled a direct “geographical” comparison of the microbiota in these two niches. Significant differences in microbial communities were apparent between the 3 groups. However, overlap between the three groups, particularly between CF and donor tissue and CF tissue and CF luminal mucus was also observed. Microbial diversity was lower in CF luminal mucus compared to CF tissue, with dominance higher in luminal mucus. For both CF and donor tissue, intra- and inter-patient variability in ecological parameters was observed. No relationships were observed between ecological parameters and CF-CT score, or features of end-stage lung disease. The end-stage CF lung is characterised by a low diversity microbiota, differing within and between individuals. No clear relationship was observed between regional microbiota variation and structural lung damage.

## Introduction

Aerobic microbiological culture-based methods indicate that a small number of ‘typical’ CF pathogens, for example, *Pseudomonas aeruginosa, Staphylococcus aureus* and *Burkholderia cepacia* complex are responsible for chronic pulmonary infection and associated decline in lung function (Sherrard et al., 2014; Flume et al., 2018). However, studies using extended culture and culture-independent DNA-based techniques, have identified that the CF lower airways contain diverse polymicrobial communities in addition to bacteria commonly reported by routine culture. Such microorganisms, frequently overlooked by routine culture, include various members of *Streptococcus* spp. and *Rothia* spp. and strict anaerobes, such as *Prevotella* spp., *Veillonella* spp. and *Actinomyces* spp. (Zhao et al., 2012; Boutin et al., 2015; Carmody et al., 2015; Muhlebach et al., 2018; Sherrard et al., 2019).

A number of previous studies have shown that a reduction in community diversity and an increase in dominance with known pathogens is associated with a decrease in lung function and disease progression (Zhao et al., 2012; Acosta et al., 2018; Sherrard et al., 2019; Cuthbertson et al., 2020). Moreover, in CF patients with end-stage disease, airway microbial communities are almost completely dominated by ‘typical’ CF pathogens, with many ‘atypical’ community members, such as anaerobes absent (Goddard et al., 2012; Maughan et al., 2012). These studies primarily investigated oropharyngeal samples, expectorated sputum or bronchoalveolar lavage (BAL) fluid (Goddard et al., 2012). Analysis of tissue samples has been based on dissected lung parenchyma from 10 patients with CF (Goddard et al., 2012), peripheral lung tissue from 31 patients with CF and non-CF bronchiectasis (Maughan et al., 2012) or limited to samples from one or two patients (Willner et al., 2012; Brown et al., 2014). It has also been shown, that in patients with idiopathic pulmonary fibrosis (IPF), bacterial load is higher in airways compared with parenchymal samples. However, no microbial community differences were found between parenchymal tissue samples from different IPF lobes. Moreover, presence of a community with a high bacterial load and low diversity in IPF patients was associated with worse clinical outcomes (Valenzi et al., 2021).

We hypothesised that regional differences in the lung microbiota are related to CT evidence of lung damage. Multiple tissue and associated airway luminal mucus samples were extracted from the lungs of CF patients at transplant with tissue samples also extracted from unused donor lungs as control. Microbial community composition was determined by Illumina MiSeq sequencing and three key questions investigated. 1) Is there intra-and inter-patient variability in microbiota composition within the airways of people with end-stage CF and does it differ from that in donor lungs? 2) In CF patients, does the microbiota in luminal mucus differ from that in lower airway tissue? and 3) Is the microbiota in end-stage CF lung disease spatially heterogeneous and related to localized structural damage.

## Methods

### Patient Enrolment and Sample Collection

The lungs of 11 patients with end-stage CF lung disease (FEV_1_ <40% predicted), undergoing transplantation (June 2010-March 2014) at the University Hospital of Gasthuisberg, Leuven, Belgium, were collected. Following IRB approval (S52174), informed consent was obtained prior to surgery. Four unused donor lungs were used as controls and these were obtained post-mortem from non-smokers without evidence of chronic respiratory disease. These donor lungs are collected under Belgian law allowing the use of declined donor organs for research purposes, which was IRB approved (S52174, S59648, S61653). Reasons for ineligibility for transplantation are outlined in **Table S1**. Patient characteristics, clinical and pre-transplant culture data were obtained from patient notes.

### Lung Tissue Sampling and Sample Preparation for MiSeq Sequencing

Processing and sampling of lung specimens was performed according to a previously published protocol (McDonough et al., 2011; Verleden et al., 2014; Boon et al., 2016). Following excision of the left lung, the main stem bronchus was cannulated, and the lung inflated to total lung capacity by applying a constant pressure of 30 cm H_2_O. Following this, lung volume was maintained, but the distending pressure was slightly reduced to a pressure of 10 cm H_2_O in order to prevent dissection of air into the interstitial space. The lungs were then frozen in liquid nitrogen vapour and stored at -80°C.

After MDCT scanning, lungs were cut from apex to base with a band saw in 2 cm thick slices. Subsequently, cores with a diameter of 1.4 cm were excised from random locations (up to 12 cores per slice) within each slice using either a sterile sharpened steel cylinder (i.e. a cork bore) or a power-driven hole-saw. The exact anatomical position of each core was recorded by photographing each slice before and after the sampling procedure. To minimise potential cross contamination between samples obtained from the same slice, extensive cleaning was performed between procedures with all tools used cleaned with disinfectants and/or sterilized. Furthermore, we removed the top layer from either side of each core with sterile surgical scalpels to minimize the use of tissue that had been exposed during sample preparation. Tissue cores were stored at -80°C until micro-CT scanning and subsequent shipment to Belfast for microbiota analysis.

### Lung Homogenate Preparation and Extraction of Bacterial DNA

Prior to processing, descriptive notes were made on each tissue core documenting size, shape, colour, number of visible bronchioles and presence of blood/luminal mucus.

Sample processing for lung homogenate preparation was performed as follows: lung tissue samples (2 cm by 1.4 cm core) were dissected into small pieces using sterile scissors and forceps and transferred into pre-sterilised ceramic homogenisation bead tubes (2.8mm, Cambio ltd., Cambridge, UK). One millilitre of sterile quarter-strength Ringer’s solution (QSRS) was added to aid homogenisation, followed by vortex mixing and sonication for 5 minutes, with subsequent bead-beating at 6.5 m/s for 45 seconds. The resulting homogenate was passed through a sterile 70 μm nylon mesh strainer (Fisher Scientific, Waltham, Massachusetts, USA), placed in a sterile 50mL falcon tube (Sarstedt, Nümbrecht, Germany), aided by gentle centrifugation at 1 RPM for 3 minutes and the application of pressure on the strainer using a sterile plastic microbiological loop. The final homogenate was stored at -80°C until enrichment of bacterial DNA. Enrichment of bacterial DNA from lung tissue homogenate was performed using a QIAamp DNA microbiome kit (Qiagen, Hilden, Germany) as per the manufacturers’ protocol.

In order to monitor for potential operator and/or environmental contamination and background DNA present in reagents/consumables, process controls were included with each batch of samples processed. This control consisted of a sterile QSRS wash of sterile scissors/forceps being transferred to a homogenisation tube, as per above, and then processed as per all subsequent steps of homogenate preparation/bacterial DNA enrichment.

### Pre-Lysis and DNA Extraction From Lower Airway CF Luminal Mucus Samples

During processing of lung tissue cores, any luminal mucus present was removed and processed independently. Therefore, luminal mucus samples were from the area where the lung tissue was derived. The first step in pre-lysis involved addition of 100 μL of 10% Sputolysin (prepared in sterile, nuclease-free, DEPC-treated water) to the luminal mucus sample. This was followed by thorough vortex mixing and incubation at room temperature for 30 minutes in a heated shaker at 2000 RPM (Eppendorf ThermoMixer C, Eppendorf Ltd., Stevenage, UK). Next, 200 μL of a 5 mg/mL solution of lysozyme (Sigma Aldrich, Dorset, UK), prepared in Roche bacterial lysis buffer (Roche Diagnostics Ltd., Burgess Hill, UK), was added to each sample, vortex-mixed and incubated for 30 minutes at 37°C in a heated shaker at 2000 RPM. Samples were then transferred to 0.1 mm glass bead tubes (Qiagen, Hilden, Germany), with bead-beating undertaken on a Fast-Prep 24 rotor stator homogeniser (MP biomedicals LLC., Santa Ana, USA) on speed 6 m/s for 40 seconds. All tubes were then centrifuged at 13,000 xg for 1 minute, followed by addition of 32 μL of 20 mg/mL proteinase K (Qiagen, Hilden, Germany). This was followed by further vortex mixing and incubation at 65°C for 10 minutes in a heated shaker (1500 RPM). Subsequent to this, 150 μL of sterile DEPC-treated, nuclease-free water (Life Technologies Ltd., Paisley, UK) was added to each tube, followed by further bead-beating on the Fast-Prep 24 homogeniser at 6 m/s for 40 seconds. This was then followed by further incubation at 95°C, for 10 minutes in a heated shaker (1000 rpm). Finally, all samples were centrifuged at 10,000 x g for 10 minutes at 4°C, with 200 μL of supernatant transferred to a fresh, sterile 1.5 mL microcentrifuge tube and stored at -80°C until DNA extraction.

The final DNA extraction and purification of all samples was performed on the MagNA pure 96 (Roche Diagnostics Ltd., Burgess Hill, UK) automated DNA extraction platform with an input volume of 200μL pre-lysed luminal mucus, using a DNA and viral NA small volume kit (Roche Diagnostics Ltd., Burgess Hill, UK). This resulted in an output volume of 100 μL of purified DNA extract.

In line with process controls implemented for lung tissue extractions, each batch of luminal mucus samples were processed alongside a control where luminal mucus was replaced by 100 μL of sterile, DEPC-treated, nuclease-free water. Controls were treated with all processing and extraction steps as per luminal mucus samples and were sequenced regardless of whether amplification of the sample occurred during sequencing library preparation or qPCR.

### Library Preparation for Illumina MiSeq Amplicon Sequencing

Sample processing and library generation was performed using an optimized version of a previously published 4-step PCR protocol described by Lundberg and colleagues (Lundberg et al., 2013). Further information on primers used for library generation is shown in **Table S6**.

### Illumina MiSeq Sequencing

Sequencing was performed on the Illumina MiSeq platform using 500-cycle V2 reagent kits (Illumina UK, Cambridge, UK). Libraries were loaded at 12.5 pM, with a 20% spike of PhiX control library (Illumina UK, Cambridge, UK) to monitor run success. Paired-end 251 BP reads were generated using the GenerateFASTQ workflow and the FASTQ-only analysis format.

### Post-Sequencing Data Processing

Paired-end Illumina MiSeq sequences were processed using QIIME (Quantitative Insights into Microbial Ecology; version 1.9.1) (Caporaso et al., 2010b). Following joining of the combined forward and reverse reads, sequences were demultiplexed, removing barcode sequences, as well as Nextera adapters and low-quality reads. Sequences were clustered into their representative operational taxonomic units (OTUs) based on 97% sequence identity *via* the UCLUST algorithm (Edgar, 2010). Sequences were then aligned against full length 16S rRNA marker gene sequences from the GreenGenes reference database (version 13.8) (DeSantis et al., 2006) *via* PyNAST (Caporaso et al., 2010a) and assigned taxonomic identities according to the Ribosomal Database Project Classifier Tool (v 2.2) (Wang et al., 2007) using open-reference OTU picking implemented within QIIME. A number of taxa were detected in the background of the negative controls (either process controls from sample processing, DNA extraction or DEPC water PCR negative controls). These communities generally produced very low read numbers and were removed as contaminants if present in similar abundance in both negative controls and samples. Furthermore, OTUs representing potential human sequences, Archaea, Cyanobacteria and unassigned OTUs were filtered out and treated as contaminating sequences prior to downstream analysis. Finally, the most prevalent taxa observed within the negative controls belonged to unclassified families *Comamonadaceae*, *Oxalobacteraceae* and *Methylophilaceae*, with low number of reads belonging to the main putative pathogens dominating community profiles in clinical samples (e.g. *Pseudomonas* spp. and *Streptococcus* spp.). Due to the low level count of amplicons for these organisms in the negative controls, we did not remove them from the data prior to downstream analysis. The positive control amplified consistently during library preparation and produced consistent community profiles both intra- and inter-run. Positive (anonymised sputum from our biobank) and negative controls were included to monitor both inter-run variability and potential reagent/user-introduced contaminants. Clinical and positive-control samples were compared to OTU’s observed in the negative-controls, followed by filtering of those OTU’s that resulted from the background signal or contaminants. Further information regarding OTU’s observed in all samples is included in online Supplementary File S2.

### Computed Tomography (CT) and Micro-CT Scoring

Preoperative chest CT scans from CF patients, performed when patients were referred for transplant, were allocated a CF-CT score using a validated, updated version of the Brody II scoring system (Brody et al., 2006; Wainwright et al., 2011). CT was also used to determine lung volume, density and weight using Horos software. Micro-CT scoring was performed on frozen CF tissue cores by one member of the research team (SEV); features of end-stage lung disease such as emphysema, fibrosis and obliterations of the airways were scored, 1 if present and 0 if absent (Boon et al., 2016; Verleden et al., 2019). Examples of these features on micro-CT images are shown in **Figure S1**.

### Statistical Analysis

Demographic and clinical characteristics between CF and donor cohorts were compared using the Mann-Whitney U-test. Analysis of raw sequence reads, operational taxonomical units (OTU) and data analysis was performed in QIIME 1.9.1 (Caporaso et al., 2010b) and R version 3.6.3. (https://www.r-project.org/). Taxa abundances are presented as relative abundance of normalised amplicon read counts for the sample with the lowest number of reads following pre-processing and QC of reads and negative controls (normalised to 2000 reads across all samples). Alpha-diversity (within group) indices: diversity (Shannon-Wiener index; H’) and dominance (D) were calculated in PAST v.3.20 (http://folk.uio.no/ohammer/past) and compared between groups using the Wilcoxon-Rank sum test (2 groups) or Kruskal-Wallis test (≥3 groups). Beta-diversity (between groups) was assessed using distance-based metric (Bray-Curtis distance) on centred log-ratio (clr) transformed OTU table and presented as a principle coordinates plot (PCoA) showing variance explained for the first two components or as the partitioning around group centroid clusters when comparing two or more groups. Differences between groups were evaluated by multivariate-permutational analysis (PERMANOVA, adonis function) and linear mixed effect modelling fixed for group and adjusted for a random effect by patient where multiple samples were included from each lung. Where appropriate, *P*-values were adjusted for multiple testing using the Benjamini-Hochberg (BH) method for false-discovery rate. The relationship between diversity indices and lung damage, measured by the CF-CT score, were determined using the Pearson’s correlation coefficient.

## Results

### Study Overview: Patients and Samples

Patient characteristics are presented in **Table 1** and complete metadata is presented in Supplementary File S1; CF patients were significantly younger, shorter, weighed less and had higher lung density than donor subjects. In total, 98 CF lung tissue samples [n = 11 patients; mean (range) 8.91 (6-12)], 20 CF luminal mucus samples [n = 8 patients; mean (range) 2.5 (1-5)] and 33 control donor (non-disease) lung tissue samples [n = 4 patients; mean (range) 8.25 (6-11)] were analysed.

**Table 1:** Demographics and clinical characteristics of participants in the study.

	CF	Donor	*P* ^#^
Number of patients	11	4	
Number of samples			
Tissue	98	33	
Luminal mucus	20	0	
Number of tissue samples per patient	8.91 (6-12)	8.25 (6-11)	0.5736
Sex			0.3104
Male	5 (45.5)	3 (75)	
Female	6 (54.5)	1 (25)	
Age at LTx/death	24 (19-33)	37 (29-42)	0.0022
Height, m	1.63 (1.50-1.78)	1.79 (1.65-1.85)	0.0183
Weight, kg	47 (37-60)	86 (80-90)	0.0015
Lung weight, g	615.4 (379.9-941.3)	457.5 (409.0-545.6)	0.2253
Lung volume, L	2.5 (1.6-4.1)	3.4 (3.0-4.0)	0.0604
Lung density, g/L	258.1 (166.2-382.0)	137.4 (101.5-172.0)	0.0110
Pre-LTx FEV_1_, % pred	22.73 (13-31)	–	–
Pre-LTx CT Brody score			–
Left upper lobe	14.00 (10.5-18)	–	
Left lingula	11.93 (5.5-21.5)	–	
Left lower lobe	11.89 (9-18)	–	
TOTAL	37.82 (27.25-57.25)		
F508del mutation			–
Homozygote	6 (54.5)	–	
Heterozygote	5 (45.5)	–	

Data is presented as n (%) or mean (range) for all measurements, unless otherwise stated. LTx, lung transplantation; FEV_1_, forced expiratory volume in 1 second expressed as % predicted. ^#^Comparisons between groups using the Mann-Whitney U-test.

### Analysis of Lung Microbiota Composition

Information regarding total OTU distribution, prior to removal of potentially contaminating OTUs, including positive and negative controls, is shown in Supplementary File S2.

In the year preceding transplantation, 19 organisms (P. aeruginosa, n = 7; Achromobacter xylosoxidans, n = 4; S. aureus, n = 3; Stenotrophomonas maltophilia, n = 2; Serratia marcescens, n = 1; Escherichia coli, n = 1; Moraxella catarrhalis, n = 1) were detected in the 11 CF patients by routine clinical sputum culture (**Table S2**). Eighteen (94.7%) of these organisms were detected in tissue samples by sequencing and for patients with luminal mucus samples, 12/15 (80%) genera were detected by both routine clinical culture and sequencing. Of the 4 control donors, 2 were culture negative; 6 organisms (M, catarrhalis, n = 2; Klebsiella oxytoca, n = 1; Streptococcus pneumoniae, n = 1; E. coli, n=1; S. aureus, n = 1) were detected by routine clinical culture in the remaining 2 donors with 5/6 (83%) of these organisms also detected by sequencing in tissue samples. All donors received prophylactic antibiotics in the 48 hours preceding explanation (**Table S1**).

**Figure 1:**
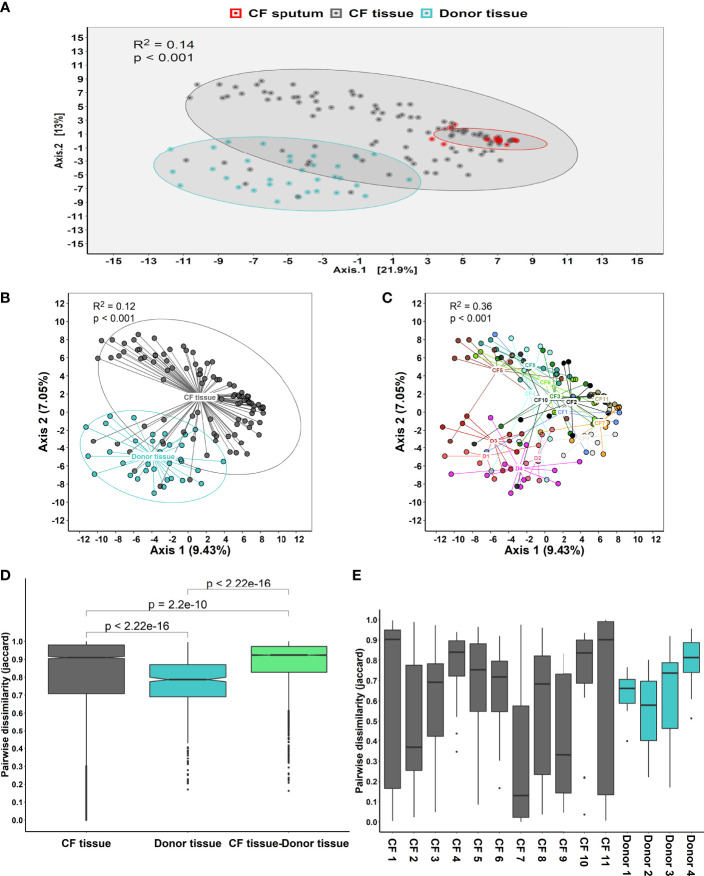
**(A)** Principal coordinate analysis (PCoA) plot between the three sample cohorts, based on the Euclidean distance metric, adjusted for repeated sampling by patient (adonis analysis [permutational multivariate ANOVA] F=11.55; R^2 =^ 0.1306; p=0.001; confidence based on 90% CI). **(B)** Analysis of multivariate homogeneity (PERMDISP) tests between CF and donor tissue cohorts to assess significant differences in median distance to centroid. **(C)** Analysis of multivariate homogeneity (PERMDISP) tests between individual patients to assess significant differences in median distance to centroid. Distances to the centroids on the first two PCoA axes showing 90% confidence interval. *P*<0.05 denotes statistical significance with 999 permutations. **(D)** Pairwise intra- and inter-dissimilarity (Jaccard’s dissimilarity) between tissue cohorts. **(E)** Intra-dissimilarity (Jaccard’s dissimilarity) for samples belonging to individual patients.

After controlling for the effects of repeated sampling for individual lungs, dispersion by sample cohort accounted for ~14% of community variance (R^2^ = 0.14, *P* < 0.001) (**Figure 1A**). Visual inspection of the principal coordinate (PCoA) plots indicated that CF tissue and luminal mucus samples overlapped to a greater extent than CF and donor tissue. However, a degree of overlap was observed between a small subset of CF and donor tissue samples (**Figures 1B, C**). There was significant intra- and inter-patient variability, with samples from the same patient in general more similar to each other than samples from different individuals (**Figure 1C**). Adonis analysis (Permutational Multivariate Analysis of Variance Using Distance Matrices) confirmed that cohort (Donor tissue vs CF tissue) had a significant effect on microbiota composition (**Figure 1B**, R^2^ = 0.12, *P* < 0.001). In addition, the microbiota differed significantly based on each individual (**Figure 1C**, R^2^ = 0.36, *P* < 0.001), with higher variation observed between individuals within the CF cohort compared to the donor cohort.

Furthermore, there was a significant dissimilarity (Jaccard’s dissimilarity) observed between the CF tissue and donor tissue samples, with higher dissimilarity observed within the CF tissue cohort (*P* = 2.2x10^-16^). Additionally, dissimilarity between each of the tissue cohorts was significantly higher compared to dissimilarity within each of the tissue cohorts on their own (CF tissue: *P* = 2.2x10^-10^ and donor tissue: *P* = 2.2x10^-16^, respectively) (**Figure 1D**). Finally, comparing tissue samples from both CF and donor cohorts confirmed that for a number of individuals, samples belonging to the CF tissue cohort demonstrated higher similarity compared to samples belonging to the donor tissue group (**Figure 1E**).

### Comparison of Lung Microbiota Composition in CF and Donor Tissue

**Figure 2:**
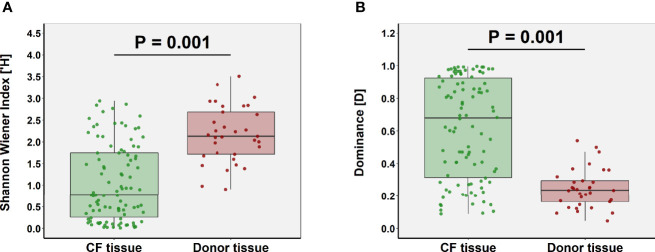
Comparison of ecological parameters in CF (n=98 samples) and donor tissue (n=33 samples). **(A)** Shannon-Wiener diversity **(B)** dominance.

**Figure 3:**
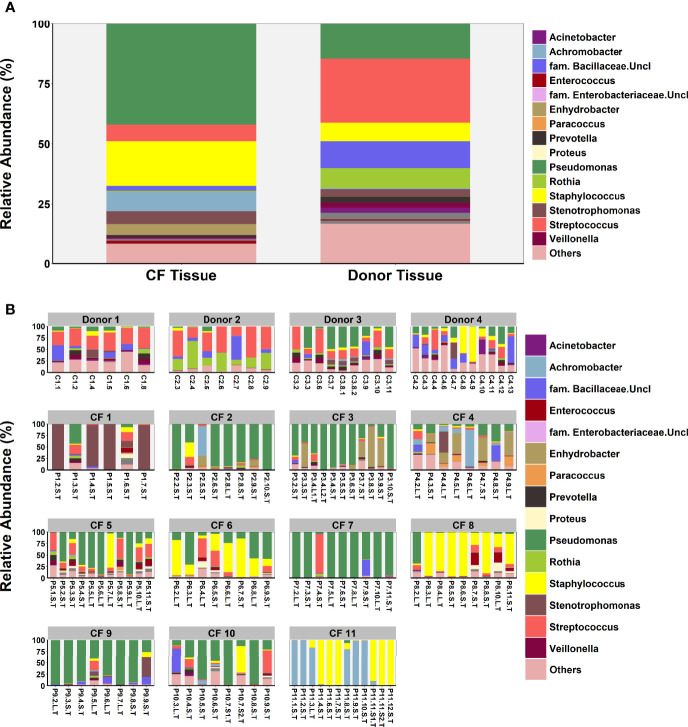
Comparison of microbial community composition in CF (n=98 samples) and donor tissue (n=33 samples). **(A)** Mean relative abundance by cohort of all genera present >1% RA. **(B)** Mean relative abundance for individual patient samples of all genera present >1% RA. Sample names are denoted as: Px.x, patient/lung-slice number (from apex to base); (Px.x.) S/L, small or large airway; (Px.x.S/L.) S/T, luminal mucus or tissue.

Microbial diversity was significantly lower in CF tissue with dominance significantly higher (**Figure 2**). Significant differences in relative abundance were identified between the two groups for 80 taxa (BH-adjusted for multiple testing; **Table S3**) with the mean relative abundance of members such as *Pseudomonas* (41.1% vs. 13.9%; *P* = 0.040) significantly greater in CF tissue (**Figure 3A**). In contrast, mean relative abundance of *Streptococcus* (25.6% vs. 6.9%; *P* = 7.1x10^-7^), *Rothia* (8.0% vs. 0.6%; *P* = 2.0x10^-7^), *Prevotella* (2.5% vs. 0.9%; *P* = 0.022) and *Veillonella* (2.1% vs. 0.4%; *P* = 2.3*10^-4^) were significantly greater in donor tissue (**Figure 3A**). There was no significant difference in mean relative abundance of *Staphylococcus* (CF, 18.2%; donor, 7.3%; *P* = 0.299)*, Achromobacter* (CF, 7.9%; donor, 0.4%; *P* = 0.810) and *Stenotrophomonas* (CF, 5.2%; donor, 3.0%; *P* = 0.072). Further information on taxa contribution in CF and donor tissue samples for individual patients are shown in **Figure 3B**.

### Comparison of Lung Microbiota Composition in Matched CF Tissue and Luminal Mucus Samples

**Figure 4:**
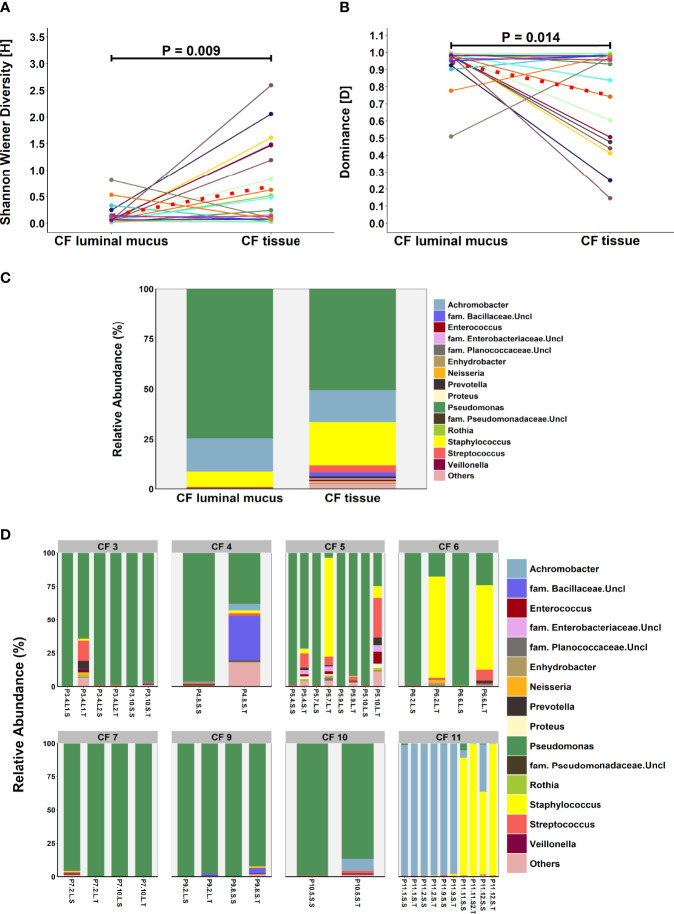
Comparison of microbial community composition in matched CF tissue and luminal mucus samples (n=20) **(A)** Shannon-Wiener diversity **(B)** dominance **(C)** mean relative abundance of all genera present >1% RA. **(D)** relative abundance for individual matched patient samples of all genera present >1% RA. Sample names are denoted as: Px.x, patient/lung-slice number (from apex to base); (Px.x.) S/L, small or large airway; (Px.x.S/L.) S/T, luminal mucus or tissue. For panels **(A, B)**, the red dotted line denotes difference in group-means.

An exploratory paired analysis of all 20 matched CF tissue and luminal mucus samples (n=8 patients) revealed that community diversity was significantly lower and dominance significantly higher in luminal mucus (**Figures 4A, B**). Significant differences in mean relative abundance were identified between the two groups for 30 genera (**Figure 4C**) with relative abundance of *Pseudomonas* (74.5% vs. 49.7%; *P* = 0.014) significantly greater in luminal mucus (**Table S4**). In contrast, the relative abundance of *Staphylococcus* (7.5% vs. 21.2%; *P* = 0.001) and *Streptococcus* (0.4% vs. 4.0%; *P* = 0.045) was significantly less in luminal mucus. The mean relative abundance of the top 10 taxa in the 20 matched samples is shown in **Figure S2**, with relative abundance of all genera present shown in **Figure 4D**.

### Intra- and Inter-Patient Variability in Lung Microbiota Composition

**Figure 5:**
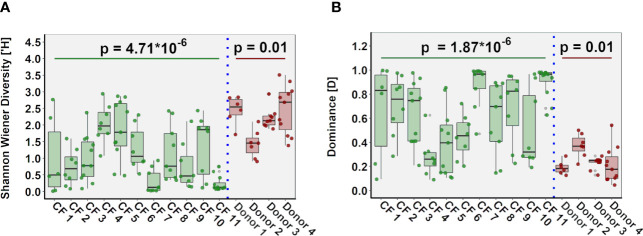
Inter-individual variation in ecological parameters in CF (n=11 patients) and donor (n=4 patients) cohorts. **(A)** Shannon-Wiener diversity **(B)** dominance.

In both cohorts, microbial community composition varied significantly within tissue samples from the same patient, while in other patients, we observed significant similarities between samples from different regions of the same lung (**Figure S3**). Inter-patient variability in community diversity and dominance was also observed (**Figure 5**). The relative abundance of genera including *Pseudomonas, Achromobacter, Staphylococcus, Stenotrophomonas, Prevotella, Veillonella* and *Porphyromonas* differed significantly between CF patients (all *P* < 0.05), with the abundance of genera including *Rothia, Streptococcus, Prevotella, Veillonella* and *Pseudomonas* significantly different between control donors (all *P* < 0.05). Furthermore, multivariate homogeneity of group dispersions/variances demonstrated significant difference between lung tissue samples from different individuals for both CF and donor tissue (R = 0.425, *P* = 0.001; Adonis test on Bray-Curtis distances; **Figure S4**).

Finally, when we compared microbiota composition and community metrics in lung slices ranging from slice 2 (apex) to slice 11 (base) of the lungs, no statistically significant differences were observed in either diversity or dominance for either cohort (**Figure S5**). Moreover, no clear trend was observed in abundance of any particular genus and location in the lungs for either group.

### Relationship Between Microbial Community Composition and Structural Damage in the End-Stage CF Lung

No significant relationships were observed between community metrics for the upper, middle and lower lobe and lung damage, measured by the CF-CT score, in those corresponding regions (*P* > 0.05; Pearson correlation). Similarly, no significant differences were apparent for community metrics in samples in which features of end-stage lung disease (fibrosis, emphysema and obliterations) were present or absent (**Figures S6A–C**). When samples were classified by the presence or absence of fibrosis, emphysema and obliterations in an exploratory analysis, there were significant differences in mean relative abundance for 3, 3 and 10 taxa, respectively (**Table S5**). Most notably, mean relative abundance of *Achromobacter* was significantly higher (*P* = 0.008) when emphysema was present (21.1%) vs. absent (6.1%) and the mean relative abundance of *Pseudomonas* was significantly higher (*P* = 0.008) in samples with (47.2%) vs. without (22.8%) obliterations. However, following BH-adjustment for multiple testing, no significant difference in taxa relative abundance were apparent for any of the measures of lung damage.

## Discussion

This study is one of the largest to date examining the lower airway microbiota in CF and unused donor lungs *via* direct tissue and luminal mucus sampling. In general, low diversity microbial communities, dominated by typical CF pathogens, were detected in the majority of CF lung tissue samples supporting the results of previous studies (Goddard et al., 2012; Maughan et al., 2012; Rudkjobing et al., 2012; Kitsios et al., 2018). *Pseudomonas* was the most abundant genus detected; other typical CF pathogens including *Achromobacter, Stenotrophomonas* and *Staphylococcus* were less abundant, but when present, were usually the dominant genus in the sample. *Streptococcus* was also among the most abundant genera. Other potential commensals including anaerobic bacteria such as *Prevotella* and *Veillonella*, were prevalent in most CF patients but were detected in low relative abundance.

Similar to previous studies (Hilty et al., 2010; Charlson et al., 2011; Morris et al., 2013; Einarsson et al., 2016; Kitsios et al., 2018), lung tissue from donors generally displayed more diverse communities with *Streptococcus, Staphylococcus* and *Rothia* commonly detected and present with a higher relative abundance than in CF tissue. Despite previously being reported in the airways of healthy individuals (Erb-Downward et al., 2011), the observation of *Pseudomonas* as one of the most abundant genera in donor lung tissue was surprising. However, this was primarily due to one subject (Donor 3) displaying a particularly high *Pseudomonas* relative abundance; this subject had a history of drug/alcohol abuse, which may have contributed to the increased abundance of the opportunistic bacteria, *P. aeruginosa.* Each donor received antibiotics in the preceding 48 hours and PWCF were also treated extensively with antibiotics prior to transplantation; such treatment has been previously shown to reduce community diversity in the short-term (Carmody et al., 2015). However, the finding that tissue samples from PWCF were less diverse and dominated with a single or few taxa is likely due to the fact that PWCF receive multiple courses of antibiotics in their lifetime, with an attendant effect on the resident airway microbiota.

PCoA analysis demonstrated some overlap in the microbiota between CF and donor tissue. Moreover, ecological measures of community composition were similar in some CF and donor tissue samples. This suggests that microbial composition in some areas of the CF lungs may be similar to that present in ‘healthy’ donor lungs, highlighting the spatial distribution of bacteria within the airways of both CF patients and control donors. Donors were intubated for between 38 and 179 hours prior to donation and it is possible that intubation may have introduced some bacteria from the upper airways into the lungs, thereby increasing airway community diversity and richness.

Although expectorated sputum is routinely used to detect the presence of potentially pathogenic organisms in the lower airways (Ronchetti et al., 2018), no previous studies have determined whether sputum is an accurate surrogate for microbial community composition in the deeper airways. Indeed, studies of infection in chronic airway diseases primarily focus on known pathogens with less emphasis or understanding of what role the “commensal” microbiota may play in the progression of disease. In the present study, we extracted luminal airway secretions from tissue samples and compared microbial community composition in luminal mucus and tissue. Although we were extremely careful when removing luminal mucus from lung tissue samples, we cannot guarantee complete separation of the two sample types and there was the potential for some cross-contamination. Diversity was lower and dominance higher in luminal mucus. Moreover, although *Pseudomonas* spp. was detected in both sample types, other taxa such as members of *Streptococcus* spp., *Staphylococcus* spp., *Rothia* spp. and common anaerobic taxa such *Prevotella* spp. and *Veillonella* spp. were significantly enriched in CF lung tissue versus luminal mucus samples. Numerous studies have investigated how microorganisms, typically identified as commensals, interact with classic CF pathogens and contribute to airway disease (Whiteson et al., 2014; Flynn et al., 2016; Quinn et al., 2016). Degradation of mucins (Flynn et al., 2016) and low oxygen conditions resulting in a decrease in pH (Whiteson et al., 2014) have been suggested as potential mechanisms which would be relevant to the end-stage CF lung.

These results demonstrate that community diversity in the lower airways is underestimated by airway derived samples such as sputum, while more diverse community composition may be observed attached to the surface of the luminal epithelium. However, sampling of airway secretions does enable detection of the dominant microorganism; with one exception, the dominant organism was the same in all matched samples, with the relative abundance usually higher in luminal mucus. *Pseudomonas* was the most abundant organism in 15/20 luminal mucus samples, suggesting that it may be enriched in airway secretions, where it can predominate and subsequently seed surrounding lower airway tissue, displacing the normal, diverse ‘healthy’ microbiota.

As multiple tissue samples from base to apex of the lung were analysed for each CF patient, we were able to determine both intra- and inter-patient variability in lung microbiota composition. As reported previously for studies analysing respiratory secretions (Stressmann et al., 2012; Zhao et al., 2012; Coburn et al., 2015), each patient harboured their own unique microbiota. Moreover, this individuality extended to how regionally distinct the microbiota was in different tissue samples; some patients displayed homogeneous communities throughout the lung, whilst others demonstrated communities differing with respect to both the dominant genera and/or overall diversity between different lung slices from base to apex. Previous studies in other end-stage cohorts have reported similar results, with Goddard and colleagues (Goddard et al., 2012) observing regional variation in 3/10 patients and Willner and colleagues (Willner et al., 2012) reporting that both patients’ lungs in their study harboured spatially heterogeneous communities. Moreover, Jorth and colleagues reported that clonally related *P. aeruginosa* isolates from different lung regions differed phenotypically with respect to factors such as antimicrobial resistance and motility (Jorth et al., 2015). Inter-individual microbiota variation was also observed, albeit to a lesser extent, in tissue samples from the 4 donor patients, suggesting that, even in health, the lung microbiota is unique for each individual. This is not surprising given that patients are variably exposed to a range of selective pressures that can modulate the microbiota, e.g. smoking, airway pollution, drug therapy and diet (Man et al., 2017).

As some regional variation in microbiota composition was apparent in CF airways, we determined if such differences were related to localized structural damage and disease progression. Overall, no clear relationship between community metrics and micro-CT scoring or CF-CT scoring of the upper, middle and lower lobe was observed. This may have been because micro-CT scores were similar for each core from the same patient with extensive structural lung damage apparent in all cores. Therefore, the airways at time of transplant may not be most suitable to understand these relationships; longitudinal analysis of samples and subsequent change in anatomical abnormalities and structural damage in CF patients with less advanced disease may be more informative in linking microbiota phenotype with lung damage and disease progression. Furthermore, functionality of the microbiota may be more relevant to lung damage than composition and additional ‘omics’ such as proteomics and metabolomics, may supplement compositional data at establishing these relationships. Acquisition of such data may be facilitated by the use of more advance cartographical approaches. For example, two studies, from the same group, have described how generation of three-dimensional (3D) microbiome and metabolome maps of explanted lungs from PWCF can be used to better understand functional interactions in the airways. These studies reported significant variation in pathogens, host-derived molecules, microbial metabolites and medications within and between CF lungs. Moreover, it allowed visualization of microbial community interactions, such as increased production of quorum sensing molecules in locations where *Pseudomonas* was in contact with *Staphylococcus* (Garg et al., 2017; Melnik et al., 2019).

Our study has a number of limitations. First, the low biomass in some of the tissue samples was a technical challenge with the potential for background contaminants having an impact on the interpretation of the results. We accounted for potentially contaminating OTUs by using a number of negative technical controls and by filtering out OTUs considered as belonging to the technical background prior to downstream analysis. Moreover, direct sampling of lung tissue provided more specific characterisation of the microbiota in distinct anatomical regions of the lung than would be achieved by sampling respiratory secretions such as expectorated or induced sputum. Our study also focused solely on bacterial community composition and did not include fungi such as *Aspergillus* and viruses, which may be present within the CF airways (Delhaes et al., 2012; Flight et al., 2014; Schwarz et al., 2018). However, fungi were not detected in any patient by routine sputum culture in the 12 months preceding transplantation suggesting that, if present, they were not a major component of the airway microbiota. Access to explanted lungs from non-diseased controls was limited to four lungs; a larger number of donor lungs would have enabled a more meaningful comparison with CF lungs but obtaining such controls is extremely challenging.

## Conclusions

The end-stage CF lung is characterised by a low diversity microbiota, dominated by typical CF pathogens which differs markedly between individual CF patients and from the more diverse microbiota present in donor lung tissue. Furthermore, although intra-patient regional variation with respect to individual genera and overall diversity was observed, there was no clear relationship between such variation and structural lung damage.

## Data Availability Statement

The datasets presented in this study can be found in online repositories. The names of the repository/repositories and accession number(s) can be found below: https://www.ebi.ac.uk/ena, PRJEB36365.

## Ethics Statement

Ethics approval was granted under Belgian law allowing the use of declined donor organs for research purposes, which was IRB approved (S52174, S59648, S61653). The patients/participants provided their written informed consent to participate in this study.

## Author Contributions

MT, JE, GE, DG, BV, and SV conceived and designed research. RV, GV, DR, and LD collected samples and clinical data. CS, GE, AL, SV, MB, and BV performed research. GE, CS, MT, and SV analyzed data. GE: bioinformatic analysis. CS, MT, GE, JE, SV, and BV wrote the paper. All authors contributed to the article and approved the submitted version.

## Funding

GE is supported by the iABC consortium, funded by the European Union Innovative Medicines Initiative (Grant Agreement number 115721) and EFPIA (iABC consortium members Novartis and Polyphor). MT and JE are supported by the Health and Social Care Northern Ireland Research and Development office (COM4978/14). SV, GV, and BV are supported by FWO (12G8718N and G083818N). SV, BV, and GV are also supported by KU Leuven (C24/18/073 and C24/050). GV is supported by the Broere foundation. MB and SV are supported by a Forton grant of the Koning Boudewijn Stiching. The stated funders were not involved in the study design, collection, analysis, and interpretation of data, the writing of this article or the decision to submit it for publication.

## Conflict of Interest

The authors declare that the research was conducted in the absence of any commercial or financial relationships that could be construed as a potential conflict of interest.

## Publisher’s Note

All claims expressed in this article are solely those of the authors and do not necessarily represent those of their affiliated organizations, or those of the publisher, the editors and the reviewers. Any product that may be evaluated in this article, or claim that may be made by its manufacturer, is not guaranteed or endorsed by the publisher.

## Supplementary Material

Supplementary Figure 1 Main features observed by micro-CT imaging in CF and control donor lungs.

Supplementary Figure 2 Comparison between the mean relative abundance of the top 10 genera in the 20 matched CF tissue and luminal mucus samples. *P*<0.05 denotes statistical significance.

Supplementary Figure 3 Relative abundance (RA) of all genera present >1% for all samples belonging to each individual patient. Sample names are denoted as: Px.x, patient/lung-slice number (from apex to base); (Px.x.) S/L, small or large airway; (Px.x.S/L.) S/T, luminal mucus or tissue.

Supplementary Figure 4 Difference in the compositional variance between individual lungs calculated using the “betadisper” function from the vegan package (2.4-6) in R, followed by significance testing using a permutation test. The permutation test assesses significant differences of median distance to centroid. *P*<0.05 denotes statistical significance with 999 permutations.

Supplementary Figure 5 Comparison of ecological parameters in lung slices ranging from slice 2 (apex) to slice 11 (base) of the lungs **(A)** Shannon-Wiener diversity **(B)** dominance.

Supplementary Figure 6 Comparison of ecological parameters (Shannon-Wiener diversity and dominance) in samples in which features of end-stage lung disease were present or absent on micro-CT images **(A)** fibrosis **(B)** emphysema and **(C)** obliterations. *P*<0.05 denotes statistical significance.
