# Supplementary material for: Microbial Community Composition in Explanted Cystic Fibrosis and Control Donor Lungs
Source: Front Cell Infect Microbiol. 2022 Mar 16;11:764585. doi: 10.3389/fcimb.2021.764585 (PMC8966769; doi:10.3389/fcimb.2021.764585)
Supplement: Supplementary Figure 1 — Main features observed by micro-CT imaging in CF and control donor lungs. [file DataSheet_1.zip › Table S1 .pdf]

Table S1 Characteristics of donors, cause of death and reason for decline of donor lung.

Patient No.	Age at death / yrs	Alcohol / drug abuse (Y/N)	Duration of intubation prior to sampling (hours)	Cause of death	Prophylactic Antibiotics (hours before explant)	Detected bacteria by clinical culture	Top 3 genera via NGS / mean % RA per patient	Reason for decline of donor lung
**Donor 1**	42	N	38	Trauma	Cefazolin (48)	Culture negative	1. *Streptococcus* – 34.2<br>2. fam *Bacillaceae* Unclassified – 10.3<br>3. *Lactococcus* – 6.6	Partially used for lobar transplant
**Donor 2**	29	Y	75	Trauma	Clindamycin (24)	Culture negative	1. *Streptococcus* – 43.8<br>2. *Rothia* – 29.6<br>3. fam *Bacillaceae* Unclassified – 11.8	Contusion due to trauma
**Donor 3**	37	Y	44	Cerebrovascular accident	Piperacillin/Tazobactam (<24)	*Moraxella catarrhalis*; *Klebsiella oxytoca*	1. *Pseudomonas* – 28.7<br>2. *Streptococcus* – 24.3<br>3. *Prevotella* – 4.8	Microemboli
**Donor 4**	40	N	179	Cardiac arrest	Co-amoxiclav (<24)	*Streptococcus pneumoniae*; *Moraxella catarrhalis*; *Escherichia coli*; *Staphylococcus aureus*	1. *Staphylococcus* – 16.8<br>2. fam *Bacillaceae* Unclassified – 14.4<br>3. *Pseudomonas* – 13.1	Logistics
