# Supplementary material for: Microbial Community Composition in Explanted Cystic Fibrosis and Control Donor Lungs
Source: Front Cell Infect Microbiol. 2022 Mar 16;11:764585. doi: 10.3389/fcimb.2021.764585 (PMC8966769; doi:10.3389/fcimb.2021.764585)
Supplement: Supplementary Figure 1 — Main features observed by micro-CT imaging in CF and control donor lungs. [file DataSheet_1.zip › Table S2.pdf]

Table S2 Comparison of pre-transplant routine culture (expectorated sputum) and NGS data from tissue and lower airway sputum samples.

Patient No.	Cultured organisms (luminal mucus within 12 months of Tx)	Relative abundance of cultured genus via NGS / mean % RA per patient		Top 3 genera via NGS / mean % RA per patient	
		Tissue	Luminal mucus	Tissue	Luminal mucus
**CF 1**	*Serratia marcescens* *Stenotrophomonas maltophilia*	*Serratia* - 0.06<br>*Stenotrophomonas* – 65.60	N/A	1. *Stenotrophomonas* – 65.6<br>2. *Pseudomonas* – 8.08<br>3. *Streptococcus* – 6.71	N/A
**CF 2**	*Escherichia coli*	*Escherichia* – 0.00	N/A	1. *Pseudomonas* – 72.49<br>2. *Achromobacter* – 8.00<br>3. *Enhydrobacter* – 4.66	N/A
**CF 3**	*Pseudomonas aeruginosa*	*Pseudomonas* – 63.50	*Pseudomonas* – 99.45	1. *Pseudomonas* – 63.50<br>2. *Enhydrobacter* – 21.21<br>3. *Streptococcus* – 3.49	*1.* *Pseudomonas* – 99.45<br>*2.* *Pseudomonadaceae*_unclassified – 0.38<br>*3.* *Staphylococcus* – 0.15
**CF 4**	*Pseudomonas aeruginosa* *Achromobacter xylosoxidans* *Moraxella catarrhalis*	*Pseudomonas* – 15.34<br>*Achromobacter* – 14.75<br>*Moraxella* – 0.05	*Pseudomonas* – 96.10<br>Achromobacter – 0.85<br>Moraxella - 0	1. *Enhydrobacter* -22.55<br>2. *Pseudomonas* – 15.34<br>3. *Achromobacter* – 14.75	1. *Pseudomonas* – 96.10<br>2. *Streptococcus* – 1.10<br>3. *Achromobacter* – 0.85
**CF 5**	*Pseudomonas aeruginosa* *Achromobacter xylosoxidans*	*Pseudomonas* – 41.32<br>*Achromobacter* – 0.33	*Pseudomonas* – 99.10<br>*Achromobacter* - 0	1. *Pseudomonas* – 41.32<br>2. *Streptococcus* – 17.4<br>3. *Staphylococcus* – 9.76	*1.* *Pseudomonas* – 99.10<br>*2.* *Streptococcus* 0.46<br>*3.* *Pseudomonadaceae*_unclassified – 0.39
**CF 6**	*Pseudomonas aeruginosa* *Achromobacter xylosoxidans* *Staphylococcus aureus*	*Pseudomonas* – 31.65<br>*Achromobacter* – 0.0125<br>*Staphylococcus* – 42.08	*Pseudomonas* – 99.28<br>*Achromobacter* – 0<br>*Staphylococcus* – 0.1	1. *Staphylococcus* – 42.08<br>2. *Pseudomonas* – 31.65<br>3. *Streptococcus* – 12.04	1. *Pseudomonas* – 99.28<br>2. *Pseudomonadaceae*_unclassified – 0.28<br>3. *Streptococcus* – 0.23
**CF 7**	*Pseudomonas aeruginosa*	*Pseudomonas* – 83.50	*Pseudomonas* – 97.18	1. *Pseudomonas* – 83.50<br>2. *Streptococcus* – 9.41<br>3. *Bacillaceae*_unclassified – 4.01	*1.* *Pseudomonas* – 97.18<br>*2.* *Pseudomonadaceae*_unclassified – 0.73<br>*3.* *Streptococcus* – 0.65
**CF 8**	*Staphylococcus aureus*	*Staphylococcus* – 63.42	N/A	1. *Staphylococcus* – 63.42<br>2. *Pseudomonas* – 9.36<br>3. *Streptococcus* – 6.01	N/A
**CF 9**	*Pseudomonas aeruginosa* *Stenotrophomonas maltophilia*	*Pseudomonas* – 77.03<br>*Stenotrophomonas* – 7.06	*Pseudomonas* – 99.58<br>*Stenotrophomonas* – 0.025	1. *Pseudomonas* – 77.03<br>2. *Stenotrophomonas* – 7.06<br>3. *Bacillaceae*_unclassified – 5.33	1. *Pseudomonas* – 99.58<br>2. *Pseudomonadaceae*_unclassified – 0.25<br>3. *Achromobacter* – 0.05
**CF 10**	*Pseudomonas aeruginosa*	*Pseudomonas* – 51.24	*Pseudomonas* – 99.15	1. *Pseudomonas* – 51.24<br>2. *Streptococcus* – 9.93<br>3. *Staphylococcus* – 8.36	1. *Pseudomonas* – 99.15<br>2. *Streptococcus* – 0.45<br>3. *Pseudomonadaceae*_unclassified – 0.25
**CF 11**	*Staphylococcus aureus* *Achromobacter xylosoxidans*	*Staphylococcus* – 51.21<br>*Achromobacter* – 46.92	*Staphylococcus* – 29.92<br>*Achromobacter* – 67.22	1. *Staphylococcus* – 51.21<br>2. *Achromobacter* – 46.92<br>3. *Pseudomonas* – 0.71	1. *Achromobacter* – 67.22<br>2. *Staphylococcus* – 29.92<br>3. *Pseudomonas* – 1.61
