# Supplementary material for: Microbial Community Composition in Explanted Cystic Fibrosis and Control Donor Lungs
Source: Front Cell Infect Microbiol. 2022 Mar 16;11:764585. doi: 10.3389/fcimb.2021.764585 (PMC8966769; doi:10.3389/fcimb.2021.764585)
Supplement: Supplementary Figure 1 — Main features observed by micro-CT imaging in CF and control donor lungs. [file DataSheet_1.zip › Table S6 .pdf]

Table S6 Primer sequences for each PCR step of Illumina MiSeq 16S rRNA gene amplicon sequencing library preparation protocol.

PCR step	Primer Name	5’-3’ Nucleotide sequence
1	515F	GTGCCAGCMGCCGCGGTAA
	806R	GGACTACHVGGGTWTCTAAT
2	806R_F1	GTGACTGGAGTTCAGACGTGTGCTCTTCCGATCT NNNNN AC GGACTACHVGGGTWTCTAAT
	806R_F2	GTGACTGGAGTTCAGACGTGTGCTCTTCCGATCT NNTNNN AC GGACTACHVGGGTWTCTAAT
	806R_F3	GTGACTGGAGTTCAGACGTGTGCTCTTCCGATCT NNCTNNN AC GGACTACHVGGGTWTCTAAT
	806R_F4	GTGACTGGAGTTCAGACGTGTGCTCTTCCGATCT NNACTNNN AC GGACTACHVGGGTWTCTAAT
	806R_F5	GTGACTGGAGTTCAGACGTGTGCTCTTCCGATCT NNGACTNNN AC GGACTACHVGGGTWTCTAAT
	806R_F6	GTGACTGGAGTTCAGACGTGTGCTCTTCCGATCT NNTGACTNNN AC GGACTACHVGGGTWTCTAAT
3	515F_F1	GCCTCCCTCGCGCCATCAGAGATGTG TATAAGAGACAG NNNN NNNN GA GTGCCAGCMGCCGCGGTAA
	515F_F2	GCCTCCCTCGCGCCATCAGAGATGTG TATAAGAGACAG NNNN T NNNN GA GTGCCAGCMGCCGCGGTAA
	515F_F3	GCCTCCCTCGCGCCATCAGAGATGTG TATAAGAGACAG NNNN CT NNNN GA GTGCCAGCMGCCGCGGTAA
	515F_F4	GCCTCCCTCGCGCCATCAGAGATGTG TATAAGAGACAG NNNN ACT NNNN GA GTGCCAGCMGCCGCGGTAA
	515F_F5	GCCTCCCTCGCGCCATCAGAGATGTG TATAAGAGACAG NNNN GACT NNNN GA GTGCCAGCMGCCGCGGTAA
	515F_F6	GCCTCCCTCGCGCCATCAGAGATGTGTATAAGAGACAGNNNNTGACTNNNNGA GTGCCAGCMGCCGCGGTAA
4	SEQ_V4_F	AATGATACGGCGACCACCGAGATCTACACGCCTCCCTCGCGCCATCAGAGATGTG
	INDEX_R_BC	CAAGCAGAAGACGGCATACGAGAT XXXXXXXX GTGACTGGAGTTCAGACGTGTGCTC*

* XXXXXXXX represents unique 8 nucleotide base barcode sequence
