# Supplementary material for: Microbial Community Composition in Explanted Cystic Fibrosis and Control Donor Lungs
Source: Front Cell Infect Microbiol. 2022 Mar 16;11:764585. doi: 10.3389/fcimb.2021.764585 (PMC8966769; doi:10.3389/fcimb.2021.764585)
Supplement: Supplementary Figure 1 — Main features observed by micro-CT imaging in CF and control donor lungs. [file DataSheet_1.zip › Tables S3-S5.pdf]

Table S3 Difference in taxa (RA) between CF and Donor tissue.

Taxa	CF (RA)	Donor (RA)	p-value	p.adj (BH method)
Agrobacterium	0.010	0.153	3.40E-11	**1.70E-08**
f_Bacillaceae_Unclassified_1	1.990	10.394	4.10E-10	**7.80E-08**
f_Gemellaceae_Unclassified_1	0.154	1.161	4.80E-10	**7.80E-08**
Bacillus	0.203	1.368	1.80E-09	**2.00E-07**
Rothia	0.604	7.982	2.10E-09	**2.00E-07**
Streptococcus	6.929	25.565	8.70E-09	**7.10E-07**
Scardovia	0.003	0.067	3.10E-08	**2.10E-06**
Planifilum	0.000	0.147	1.00E-07	**6.10E-06**
f_Thermoactinomycetaceae_Unclassified_2	0.004	0.623	1.20E-07	**6.50E-06**
Ochrobactrum	0.164	1.070	1.30E-07	**6.50E-06**
f_Thermoactinomycetaceae_Unclassified_1	0.028	0.755	2.50E-07	**1.10E-05**
o_Lactobacillales_Unclassified	0.234	0.705	5.90E-07	**2.40E-05**
Moraxella	0.051	1.118	1.30E-06	**4.90E-05**
Actinomyces	0.453	1.373	3.10E-06	**1.10E-04**
Mycobacterium	0.039	0.611	3.4E-06	**1.10E-04**
f_Micrococcaceae_Unclassified_1	0.002	0.077	5.50E-06	**1.70E-04**
Veillonella	0.434	2.118	8.60E-06	**2.30E-04**
Anoxybacillus	0.005	0.065	9.40E-06	**2.40E-04**
Janthinobacterium	0.251	0.000	1.30E-05	**3.20E-04**
o_Bacillales_Unclassified_1	0.025	0.776	2.40E-05	**5.50E-04**
f_Planococcaceae_Unclassified_1	0.144	0.277	3.10E-05	**6.90E-04**
f_Oxalobacteraceae_Unclassified_1	0.022	0.108	4.30E-05	**9.10E-04**
Kocuria	0.083	0.539	5.20E-05	**1.00E-03**
f_Bacillaceae_Unclassified_2	0.031	0.077	5.50E-05	**1.00E-03**
Corynebacterium	0.296	1.441	5.60E-05	**1.00E-03**
Rhodococcus	0.144	0.297	5.60E-05	**1.00E-03**
f_Nocardiopsaceae_Unclassified_1	0.000	0.052	9.40E-05	**1.50E-03**
f_Microbacteriaceae_Unclassified_1	0.000	0.021	9.40E-05	**1.50E-03**
Prauseria	0.000	0.021	9.40E-05	**1.50E-03**
f_Ruminococcaceae_Unclassified	0.005	0.073	0.00018	**2.90E-03**
Proteus	0.865	0.000	0.00029	**4.40E-03**
Leptotrichia	0.080	0.159	0.00038	**5.50E-03**
Acinetobacter	0.397	2.067	0.00043	**6.20E-03**
Saccharopolyspora	0.000	0.044	0.00051	**6.90E-03**
Lysinibacillus	0.000	0.008	0.00051	**6.90E-03**
o_Clostridiales_Unclassified_1	0.019	0.152	0.00066	**8.60E-03**
Streptomyces	0.004	0.036	0.0007	**8.90E-03**
o_Bacillales_Unclassified_2	0.113	0.191	0.00078	**9.70E-03**
Rhodobacter	0.001	0.015	0.0008	**9.70E-03**
Phyllobacterium	0.049	0.000	0.00084	**9.80E-03**
Solibacillus	0.003	0.080	0.00085	**9.80E-03**
f_Microbacteriaceae_Unclassified_2	0.001	0.015	0.00087	**9.90E-03**
Alloiococcus	0.002	0.017	0.00096	**1.10E-02**
Bifidobacterium	0.009	0.056	0.00105	**1.10E-02**
f_Comamonadaceae_Unclassified_2	0.013	0.033	0.00188	**2.00E-02**
f_Chitinophagaceae_Unclassified_1	0.008	0.039	0.00214	**2.10E-02**
Porphyromonas	0.071	0.215	0.0023	**2.10E-02**
Actinobacillus	0.014	0.121	0.00257	**2.10E-02**
Desulfosporosinus	0.000	0.042	0.00272	**2.10E-02**
f_Paenibacillaceae_Unclassified	0.000	0.029	0.00272	**2.10E-02**
o_Legionellales_Unclassified	0.000	0.020	0.00272	**2.10E-02**
p_FBP_Unclassified	0.000	0.018	0.00272	**2.10E-02**
Cupriavidus	0.000	0.014	0.00272	**2.10E-02**
Alicyclobacillus	0.000	0.012	0.00272	**2.10E-02**
Thermoactinomyces	0.000	0.012	0.00272	**2.10E-02**
Ruminococcus	0.001	0.000	0.00272	**2.10E-02**
f_Nocardiaceae_Unclassified	0.000	0.009	0.00272	**2.10E-02**
f_Peptococcaceae_Unclassified_1	0.000	0.009	0.00272	**2.10E-02**
Planomicrobium	0.000	0.008	0.00272	**2.10E-02**
Gluconobacter	0.000	0.008	0.00272	**2.10E-02**
g_Candidatus_Protochlamydia	0.000	0.006	0.00272	**2.10E-02**
Mesorhizobium	0.000	0.006	0.00272	**2.10E-02**
Moryella	0.011	0.064	0.00305	**2.30E-02**
Hyphomicrobium	0.022	0.105	0.00308	**2.30E-02**
f_[Paraprevotellaceae]_g_Prevotella	0.119	0.605	0.00349	**2.50E-02**
Rhodoplanes	0.001	0.030	0.00352	**2.50E-02**
o_Actinomycetales_Unclassified_1	0.006	0.036	0.00354	**2.50E-02**
Gemella	0.013	0.041	0.00364	**2.60E-02**
f_Methylobacteriaceae_Unclassified	0.110	0.274	0.00384	**2.70E-02**
Sporosarcina	0.001	0.053	0.00394	**2.70E-02**
o_Burkholderiales_Unclassified_1	0.001	0.020	0.00394	**2.70E-02**
o_Gemellales_Unclassified_1	0.001	0.006	0.00424	**2.80E-02**
Mogibacterium	0.003	0.021	0.00457	**3.00E-02**
Catonella	0.004	0.008	0.0047	**3.10E-02**
Delftia	0.059	0.003	0.00595	**3.80E-02**
c_Bacilli_Unclassified	0.022	0.036	0.00616	**3.90E-02**
Pseudomonas	41.097	13.891	0.00637	**4.00E-02**
Acetobacter	0.013	0.045	0.00689	**4.30E-02**
Enterococcus	1.095	0.508	0.00809	**4.90E-02**
f_Gemellaceae_Unclassified_2	0.005	0.041	0.01046	6.30E-02
Peptostreptococcus	0.013	0.029	0.01052	6.30E-02

f_Streptococcaceae_Unclassified	0.083	0.117	0.01131	6.60E-02
o_JG30.KF.CM45_Unclassified	0.002	0.036	0.0114	6.60E-02
o_Clostridiales_Unclassified_2	0.014	0.123	0.0132	6.80E-02
Oxobacter	0.000	0.061	0.01493	6.80E-02
f_Streptosporangiaceae_Unclassified	0.000	0.027	0.01493	6.80E-02
Rummeliibacillus	0.000	0.018	0.01493	6.80E-02
Xanthobacter	0.000	0.017	0.01493	6.80E-02
f_Veillonellaceae_Unclassified_1	0.000	0.015	0.01493	6.80E-02
Aeromicrobium	0.000	0.012	0.01493	6.80E-02
Rubricoccus	0.000	0.012	0.01493	6.80E-02
o_WD2101_Unclassified	0.000	0.012	0.01493	6.80E-02
f_Legionellaceae_Unclassified_2	0.000	0.009	0.01493	6.80E-02
Thermomonas	0.000	0.009	0.01493	6.80E-02
f_Actinomycetaceae_Unclassified_2	0.000	0.008	0.01493	6.80E-02
Balneimonas	0.000	0.008	0.01493	6.80E-02
f_Exiguobacteraceae._Unclassified_2	0.000	0.006	0.01493	6.80E-02
c_Mollicutes_Unclassified	0.000	0.006	0.01493	6.80E-02
f_Kineosporiaceae_Unclassified_2	0.000	0.005	0.01493	6.80E-02
Planococcus	0.000	0.005	0.01493	6.80E-02
k_Bacteria_Unclassified_1	0.000	0.003	0.01493	6.80E-02
f_Nocardiopsaceae_Unclassified_2	0.000	0.003	0.01493	6.80E-02
f_Methylophilaceae_Unclassified_2	0.000	0.003	0.01493	6.80E-02
Pseudoalteromonas	0.000	0.003	0.01493	6.80E-02
k_Bacteria_Unclassified_2	0.000	0.003	0.01493	6.80E-02
f_Exiguobacteraceae._Unclassified_1	0.004	0.044	0.01605	7.20E-02
f_Rhodocyclaceae_Unclassified_1	0.002	0.029	0.01605	7.20E-02
Legionella	0.007	0.009	0.0166	7.40E-02
Williamsia	0.001	0.006	0.01734	7.60E-02
Megasphaera	0.003	0.012	0.01881	8.20E-02
Actinomycetospora	0.001	0.094	0.01933	8.20E-02
Cryocola	0.001	0.024	0.01933	8.20E-02
Sphingobacterium	0.006	0.232	0.01934	8.20E-02
f_Sporichthyaceae_Unclassified	0.001	0.038	0.01979	8.30E-02
f_Clostridiaceae_Unclassified_2	0.001	0.009	0.01979	8.30E-02
Enhydrobacter	4.745	0.048	0.02	8.30E-02
o_Gemmatimonadales_Unclassified	0.001	0.047	0.02124	8.60E-02
f_Intrasporangiaceae_Unclassified_2	0.002	0.012	0.02124	8.60E-02
o_Sphingobacteriales_Unclassified	0.002	0.012	0.02175	8.70E-02
f_Geodermatophilaceae_Unclassified_2	0.002	0.009	0.02175	8.70E-02
Sediminibacterium	0.019	0.086	0.02207	8.80E-02
Prevotella	0.868	2.480	0.02251	8.80E-02
Morganella	0.046	0.000	0.02265	8.80E-02
Thermicanus	0.006	0.095	0.02752	1.10E-01
f_Bifidobacteriaceae_Unclassified_1	0.021	0.000	0.02873	1.10E-01
f_Alcaligenaceae_Unclassified_1	0.023	0.002	0.02933	1.10E-01
Clostridium	0.041	0.129	0.03147	1.20E-01
Neisseria	0.323	0.777	0.03207	1.20E-01
f_Planococcaceae_Unclassified_2	0.005	0.026	0.0354	1.30E-01
Paenibacillus	0.009	0.077	0.03567	1.30E-01
f_Xanthomonadaceae_Unclassified_2	0.005	0.021	0.03854	1.40E-01
f_Enterococcaceae_Unclassified	0.008	0.014	0.03911	1.40E-01
Selenomonas	0.002	0.021	0.0406	1.50E-01
f_Clostridiaceae_Unclassified_1	0.002	0.015	0.04265	1.50E-01
Mobiluncus	0.002	0.026	0.04406	1.60E-01
Gemmata	0.003	0.011	0.04698	1.70E-01

Table S4 Difference in taxa (RA) between CF luminal mucus and tissue.

Taxa	Luminal mucus (RA)	Tissue (RA)	p-value	p.adj (BH adjusted)
Staphylococcus	7.528	21.158	**0.001**	0.086
Lactobacillus	0.003	0.115	**0.001**	0.086
f_Planococcaceae_Unclassified_1	0.003	0.215	**0.001**	0.086
Prevotella	0.020	0.693	**0.004**	0.140
Acinetobacter	0.005	0.123	**0.004**	0.140
Pseudomonas	74.505	49.710	**0.014**	0.190
Rothia	0.015	0.263	**0.009**	0.190
Enterococcus	0.003	0.505	**0.017**	0.190
Paracoccus	0.000	0.115	**0.020**	0.190
Proteus	0.000	0.305	**0.010**	0.190
f_Enterobacteriaceae_Unclassified_1	0.005	0.595	**0.017**	0.190
Bacillus	0.000	0.170	**0.020**	0.190
Ochrobactrum	0.000	0.140	**0.020**	0.190
o_Bacillales_Unclassified_2	0.010	0.175	**0.011**	0.190
Granulicatella	0.005	0.130	**0.020**	0.190
Porphyromonas	0.000	0.058	**0.020**	0.190
f_Lachnospiraceae_Unclassified_1	0.000	0.088	**0.020**	0.190
f_Streptococcaceae_Unclassified	0.000	0.050	**0.020**	0.190
Methylobacterium	0.000	0.180	**0.020**	0.190
Neisseria	0.015	0.298	**0.025**	0.230
f_Bacillaceae_Unclassified_1	0.003	1.928	**0.035**	0.270
Actinomyces	0.003	0.168	**0.032**	0.270

**o_Lactobacillales_Unclassified_1**	0.010	0.173	**0.033**	0.270
**o_Lactobacillales_Unclassified_2**	0.000	0.010	**0.040**	0.270
**Fusobacterium**	0.000	0.035	**0.040**	0.270
**Delftia**	0.000	0.015	**0.040**	0.270
**Micrococcus**	0.000	0.018	**0.040**	0.270
**Streptococcus**	0.380	3.963	**0.045**	0.290
**Enhydrobacter**	0.018	0.208	**0.047**	0.290

Table S5 Difference in taxa (RA) for samples stratified by the presence or absence of fibrosis, emphysema and obliterations.

Taxa	micro-CT Findings	p-value	p.adj (BH adjusted)
**f_Ellin6075_Unclassified**	**Fibrosis**	**0.029**	0.69
**Blautia**	**Fibrosis**	**0.029**	0.69
**Propionibacterium**	**Fibrosis**	**0.044**	0.69
**Achromobacter**	**Emphysema**	**0.006**	0.78
**f_Alcaligenaceae_Unclassified_1**	**Emphysema**	**0.015**	0.78
**o_Lactobacillales_Unclassified**	**Emphysema**	**0.040**	0.78
**Pseudomonas**	**Oblitaration**	**0.008**	0.74
**Peptoniphilus**	**Oblitaration**	**0.008**	0.74
**Mogibacterium**	**Oblitaration**	**0.018**	0.74
**f_Pasteurellaceae_Unclassified_2**	**Oblitaration**	**0.018**	0.74
**f_Chitinophagaceae_Unclassified_1**	**Oblitaration**	**0.018**	0.74
**Turicibacter**	**Oblitaration**	**0.018**	0.74
**Peptococcus**	**Oblitaration**	**0.018**	0.74
**Sphingomonas**	**Oblitaration**	**0.030**	0.74
**f_Burkholderiaceae_Unclassified_1**	**Oblitaration**	**0.035**	0.74
**Achromobacter**	**Oblitaration**	**0.046**	0.74
