# Supplementary figures and images for: Microbial Community Composition in Explanted Cystic Fibrosis and Control Donor Lungs
Source: Front Cell Infect Microbiol. 2022 Mar 16;11:764585. doi: 10.3389/fcimb.2021.764585 (PMC8966769; doi:10.3389/fcimb.2021.764585)

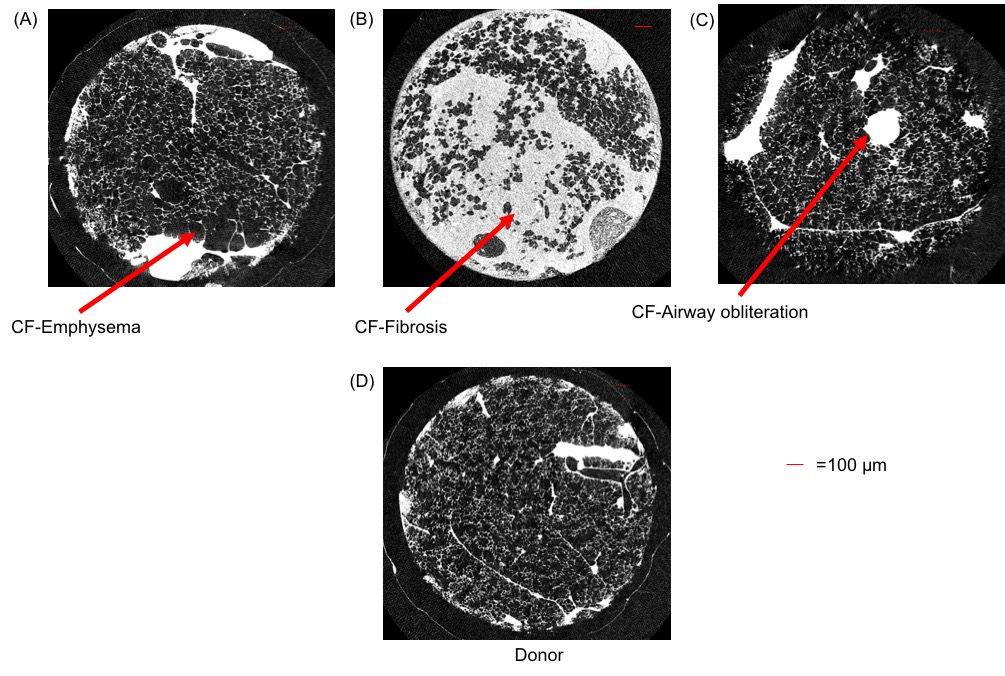

Supplement: Supplementary Figure 1 — Main features observed by micro-CT imaging in CF and control donor lungs. [file DataSheet_1.zip › Figure_S1_300dpi.tif]

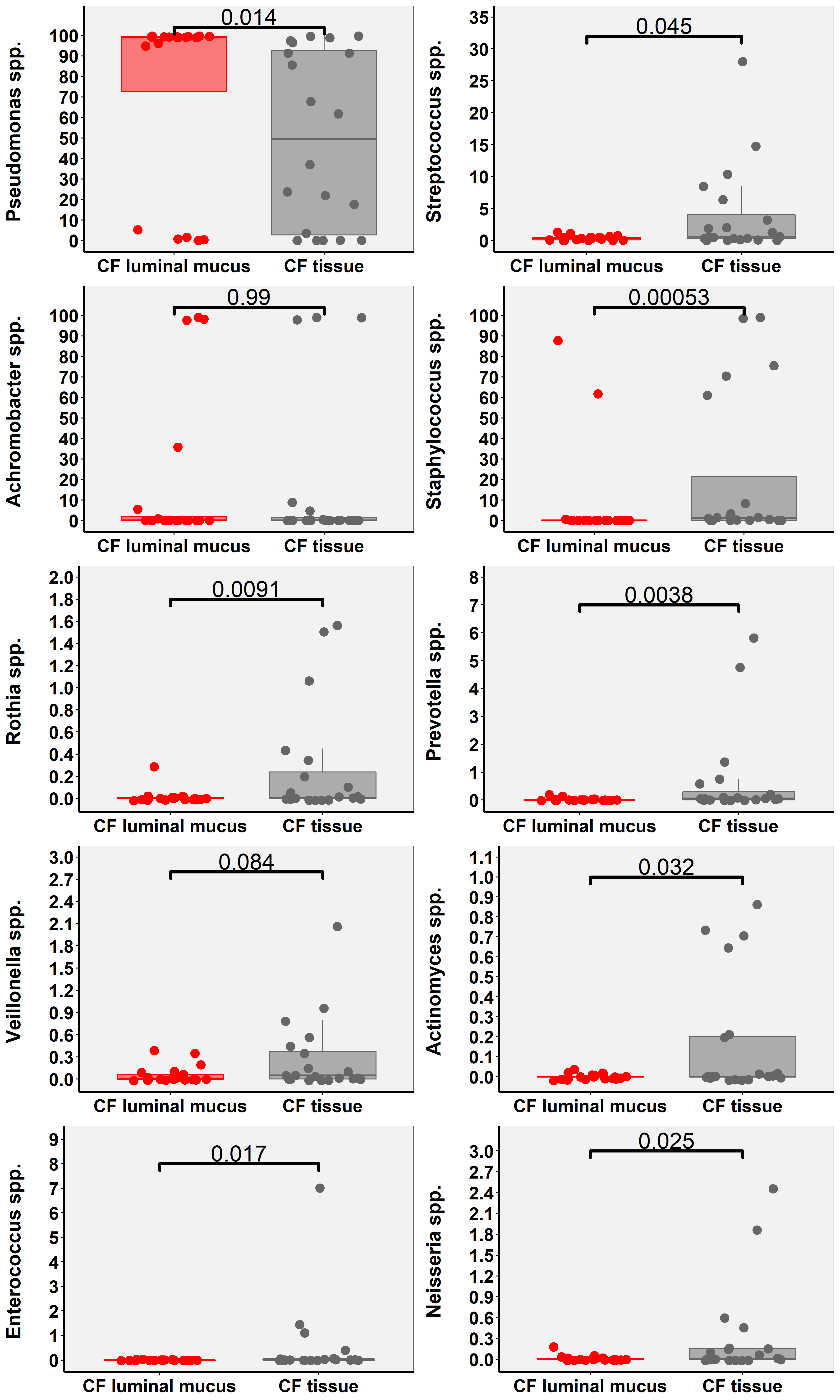

Supplement: Supplementary Figure 1 — Main features observed by micro-CT imaging in CF and control donor lungs. [file DataSheet_1.zip › Figure_S2_300dpi.tif]

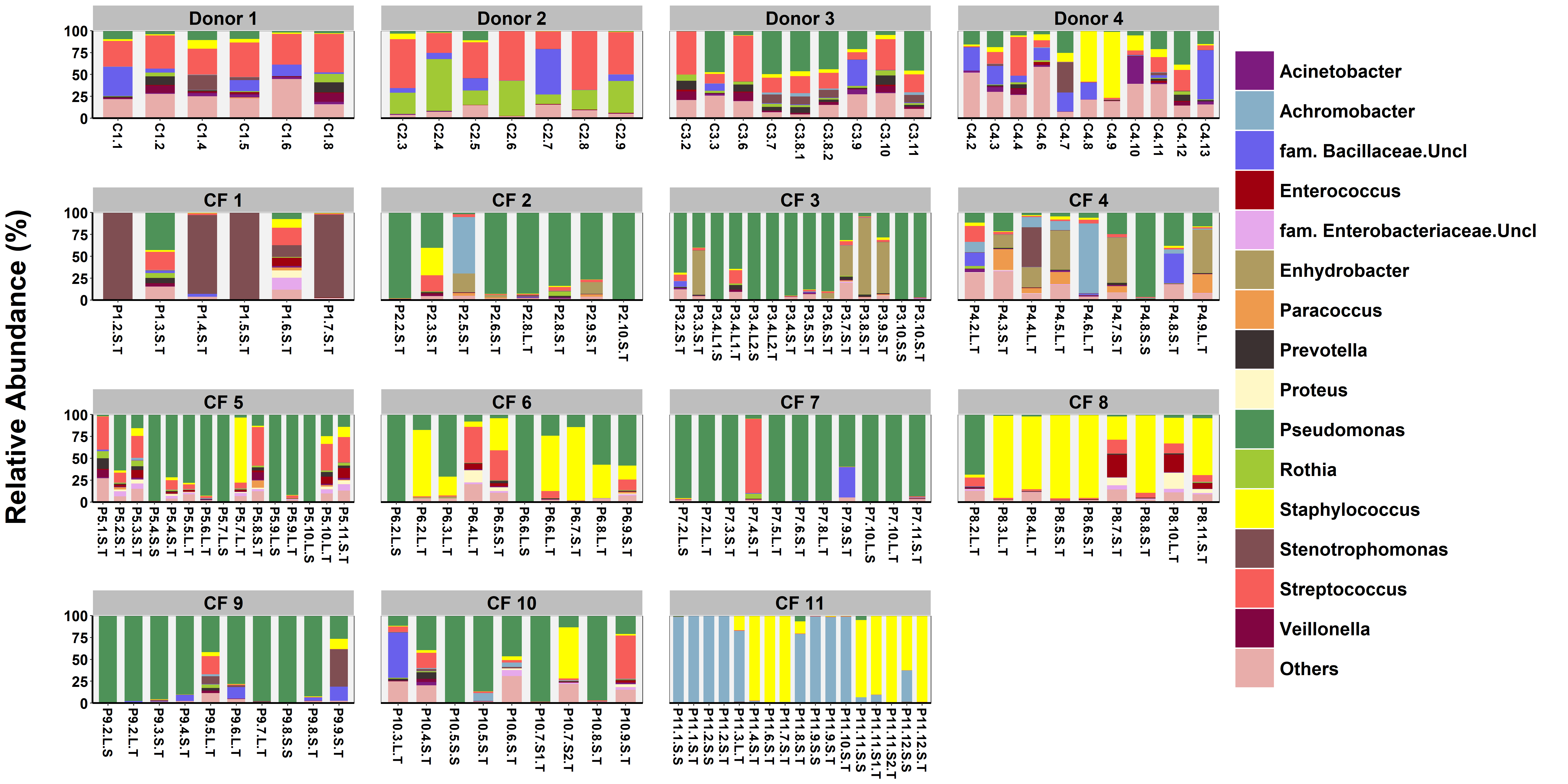

Supplement: Supplementary Figure 1 — Main features observed by micro-CT imaging in CF and control donor lungs. [file DataSheet_1.zip › Figure_S3_300dpi.tif]

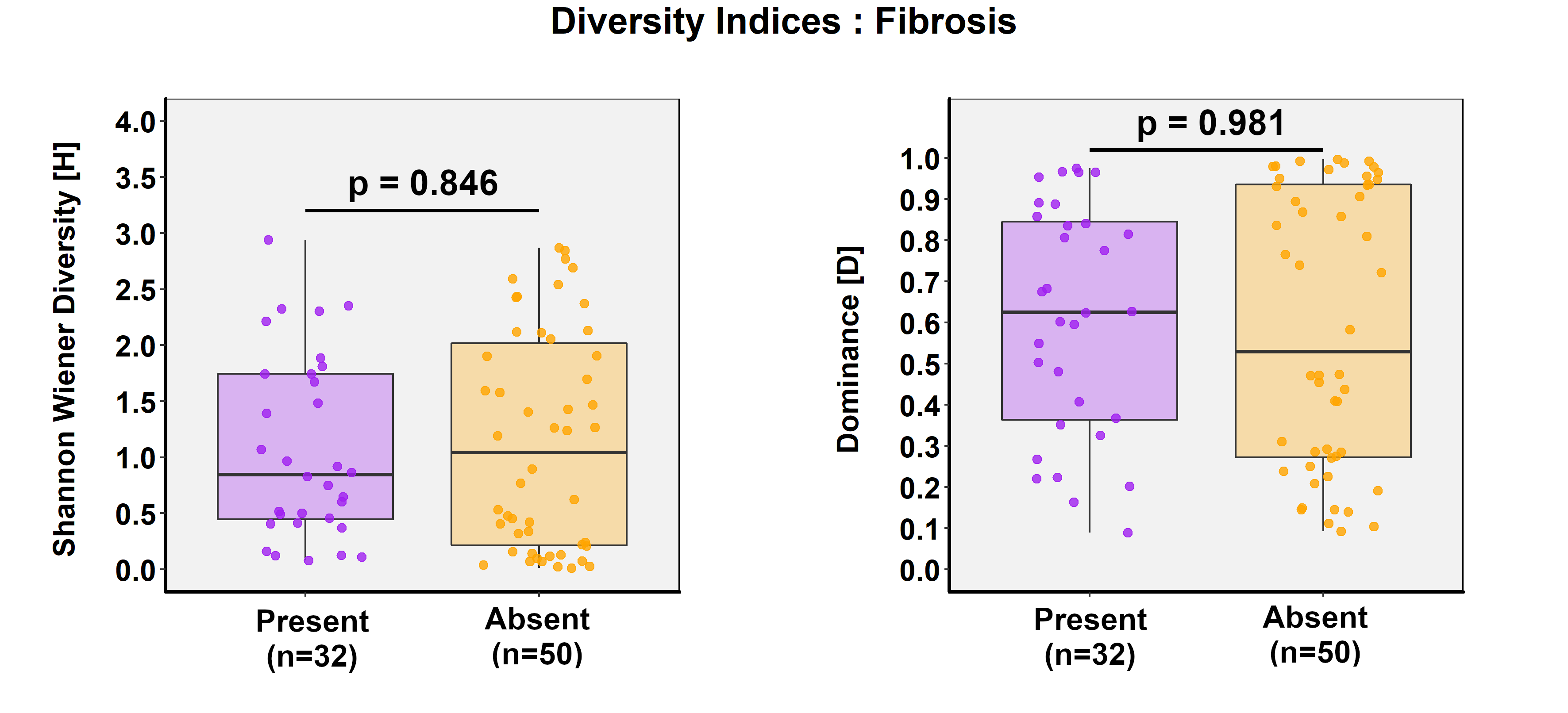

Supplement: Supplementary Figure 1 — Main features observed by micro-CT imaging in CF and control donor lungs. [file DataSheet_1.zip › Figure_S6A_300dpi.tif]

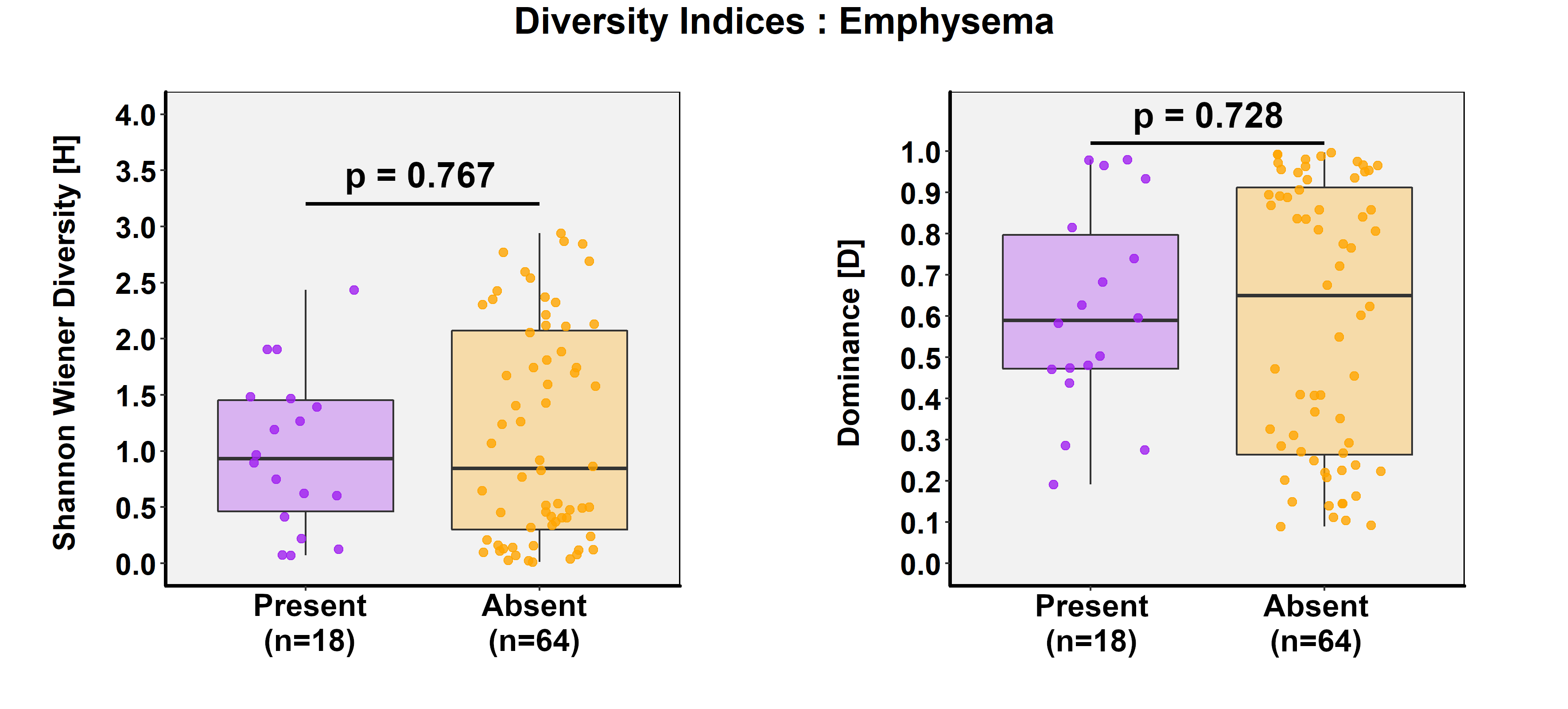

Supplement: Supplementary Figure 1 — Main features observed by micro-CT imaging in CF and control donor lungs. [file DataSheet_1.zip › Figure_S6B_300dpi.tif]

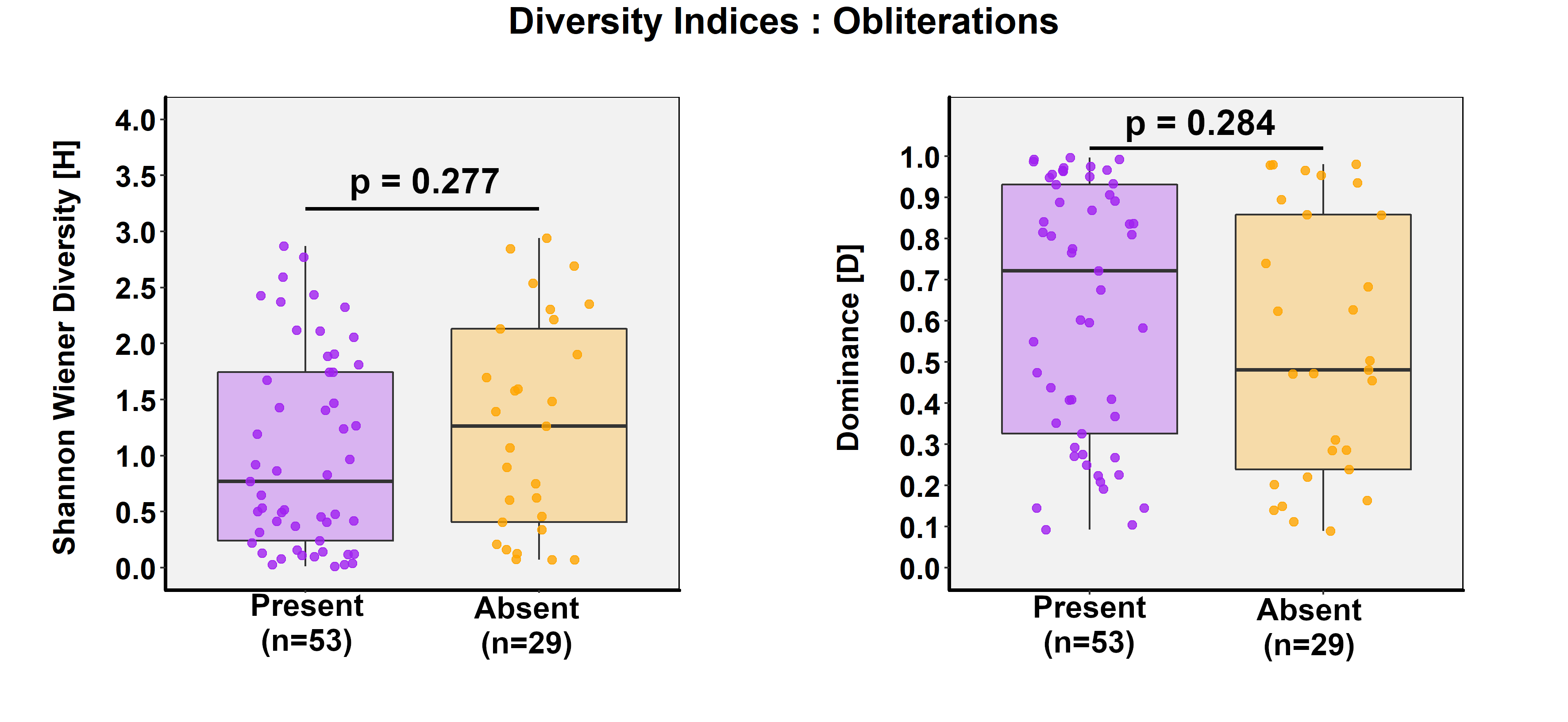

Supplement: Supplementary Figure 1 — Main features observed by micro-CT imaging in CF and control donor lungs. [file DataSheet_1.zip › Figure_S6C_300dpi.tif]
